# Total arterial revascularization on OPCABG with the exclusive use of two internal mammary arteries – a single center ten-year study analysis

**DOI:** 10.1186/1749-8090-8-S1-O198

**Published:** 2013-09-11

**Authors:** S Prapas, I Panagiotopoulos, I Linardakis, V Panagiotakopoulos, K Katsavrias, D Pavlopoulos

**Affiliations:** 1 Cardiac Surgery Dpt., Henry Dunant Hospital, Athens, Greece

## Background

Our aim is to record and present our ten-years’ experience on total arterial revascularization treatment of coronary artery disease with the exclusive use of both internal mammary arteries on OPCABG.

## Methods

From February 2001 till September 2011, 1077 patients underwent a total arterial revascularization OPCABG procedure with the use of both internal mammary arteries. The “non-touch Aorta Technique” was utilized and the “π-graft”, the “y- graft”, “t-graft”, “λ-graft” and “ψ-graft” techniques were employed. The mean age of our population was 66 years, with a male majority of 84.5%. 72 patients were diabetics (6.7%), 260 patients with LV dysfunction (EF<45%), 73 (7.2%) with renal insufficiency of which 19 under dialysis (1.8%).

## Results

The mean number of peripheral arterial anastomoses consisted of 2.8/patient. 569 patients had sequential anastomoses performed with a mean 1.35/patient. Atrial fibrillation was observed in 206 patients (19.1%). IABP was employed on 19 patients preoperatively and 14 patients postoperatively. Emergency revision was performed on 17 patients (1.6%), 14 (1.3%) for hemorrhage and 3 for hemodynamic instability (<0.3%). Sixteen patients remained under prolonged intubation >48hrs (1.6%). Postoperative neurological complications were observed in 5 patients (<0.5%) with stroke. Twenty-two patients (2%) presented sternal wound infection, 10 of which were diabetic (2 insulin and 8 non-insulin dependent). GI complications were observed in 18 patients (1.8%). Psychiatric help was assessed on 8 patients (0.7%). Long term mortality consisted of 25 and hospital mortality of 7 patients (<1%).

## Conclusions

Total arterial revascularization, with the use of both internal mammary arteries, on off pump beating heart surgery is a feasible combination with excellent results when performed by experienced cardiac surgeons.

